# Supplementary material for: Synergistic and independent roles for Nodal and FGF in zebrafish cardiac progenitor cell migration and asymmetric heart morphogenesis
Source: Development. 2025 Oct 10;152(19):dev204873. doi: 10.1242/dev.204873 (PMC12539208; doi:10.1242/dev.204873)
Supplement: Supplementary information [file develop-152-204873-s1.pdf]

Table S1. Fluorescence intensity values of *Tg(myl7:Lifeact-EGFP)* embryos.

Embryo	Condition	Side	Mean Fluorescence Value	Ratio (L/R)
1	WT	Left	1349.94	2.69
1	WT	Right	501.77	
2	WT	Left	2322.77	1.94
2	WT	Right	1199.43	
3	WT	Left	621.93	2.67
3	WT	Right	233.15	
4	WT	Left	2220.61	4.14
4	WT	Right	536.99	
5	ntl MO	Left	547.29	0.77
5	ntl MO	Right	715.64	
6	ntl MO	Left	692.61	0.79
6	ntl MO	Right	879.54	
7	ntl MO	Left	645.13	0.67
7	ntl MO	Right	968.04	
8	ntl MO	Left	1123.93	1.20
8	ntl MO	Right	934.84	
9	SB-505124	Left	811.40	0.96
9	SB-505124	Right	845.70	
10	SB-505124	Left	642.86	0.81
10	SB-505124	Right	797.09	
11	SB-505124	Left	1382.32	1.32
11	SB-505124	Right	1045.72	
12	SB-505124	Left	958.35	1.04
12	SB-505124	Right	918.12	
13	SB-505124	Left	509.50	1.86
13	SB-505124	Right	274.27	
14	spaw MO	Left	766.05	0.80
14	spaw MO	Right	953.62	
15	spaw MO	Left	3281.47	1.71
15	spaw MO	Right	1916.12	
16	spaw MO	Left	466.68	0.83
16	spaw MO	Right	564.29	
17	spaw MO	Left	1062.37	1.37
17	spaw MO	Right	774.68	
18	spaw MO	Left	645.79	0.70
18	spaw MO	Right	928.29	
19	spaw MO	Left	417.44	1.30
19	spaw MO	Right	320.33	
20	spaw MO	Left	401.35	0.84
20	spaw MO	Right	476.79	

Development • Supplementary information

Table S2. Fluorescence intensity values of phalloidin in *Tg(myl7:EGFP)* embryos.

Embryo	Condition	Side	Mean Fluorescence Value	Ratio (L/R)
1	WT	Left	270.82	1.55
1	WT	Right	174.59	
1	WT	Whole	224.57	
2	WT	Left	349.40	1.49
2	WT	Right	234.08	
2	WT	Whole	280.34	
3	WT	Left	329.57	1.23
3	WT	Right	267.16	
3	WT	Whole	293.85	
4	WT	Left	222.92	1.04
4	WT	Right	214.63	
4	WT	Whole	221.88	
5	WT	Left	262.56	1.29
5	WT	Right	203.26	
5	WT	Whole	223.11	
6	WT	Left	278.47	1.14
6	WT	Right	244.65	
6	WT	Whole	269.58	
7	WT	Left	244.45	1.47
7	WT	Right	166.11	
7	WT	Whole	216.32	
8	SU5402	Left	136.55	1.12
8	SU5402	Right	121.94	
8	SU5402	Whole	131.73	
9	SU5402	Left	196.49	1.71
9	SU5402	Right	114.59	
9	SU5402	Whole	160.20	
10	SU5402	Left	393.22	1.13
10	SU5402	Right	349.39	
10	SU5402	Whole	375.86	
11	SU5402	Left	383.40	1.31
11	SU5402	Right	293.20	
11	SU5402	Whole	357.45	
12	SU5402	Left	338.85	1.34
12	SU5402	Right	251.94	
12	SU5402	Whole	303.53	
13	SU5402	Left	336.59	1.11
13	SU5402	Right	303.46	
13	SU5402	Whole	316.90	
14	SU5402	Left	390.03	1.53
14	SU5402	Right	254.55	
14	SU5402	Whole	305.81	
15	SU5402	Left	338.41	1.20
15	SU5402	Right	283.11	
15	SU5402	Whole	309.87	

Development • Supplementary information

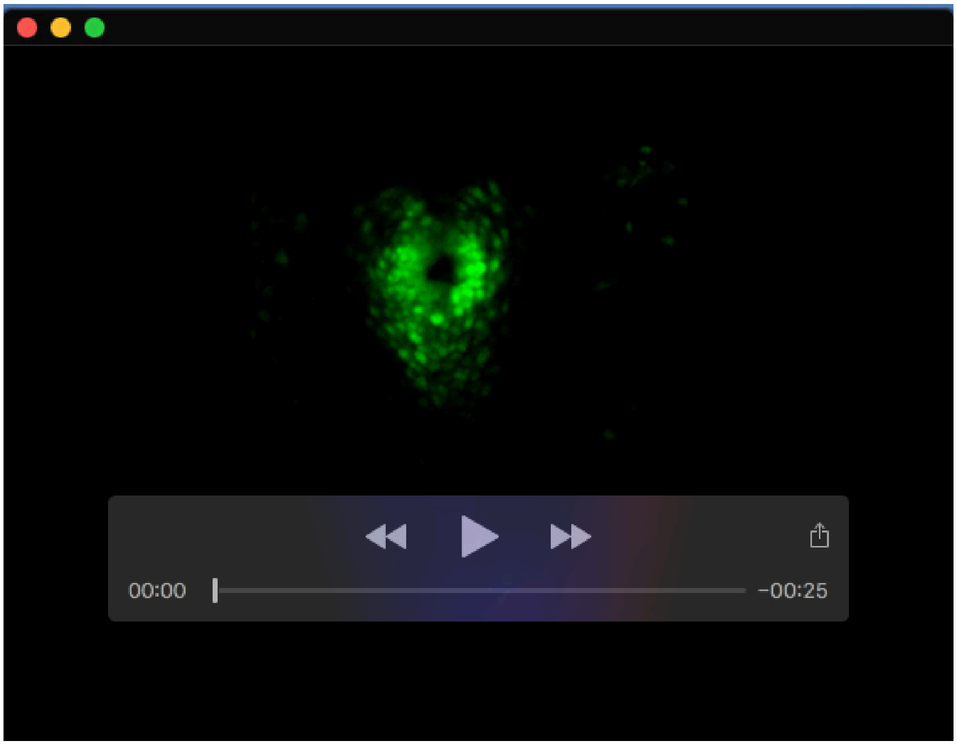

Movie 1. Live imaging of CPCs jogging in a WT transgenic *Tg(myl7:EGFP)* embryo from the onset of cardiac cone formation until three hours after.

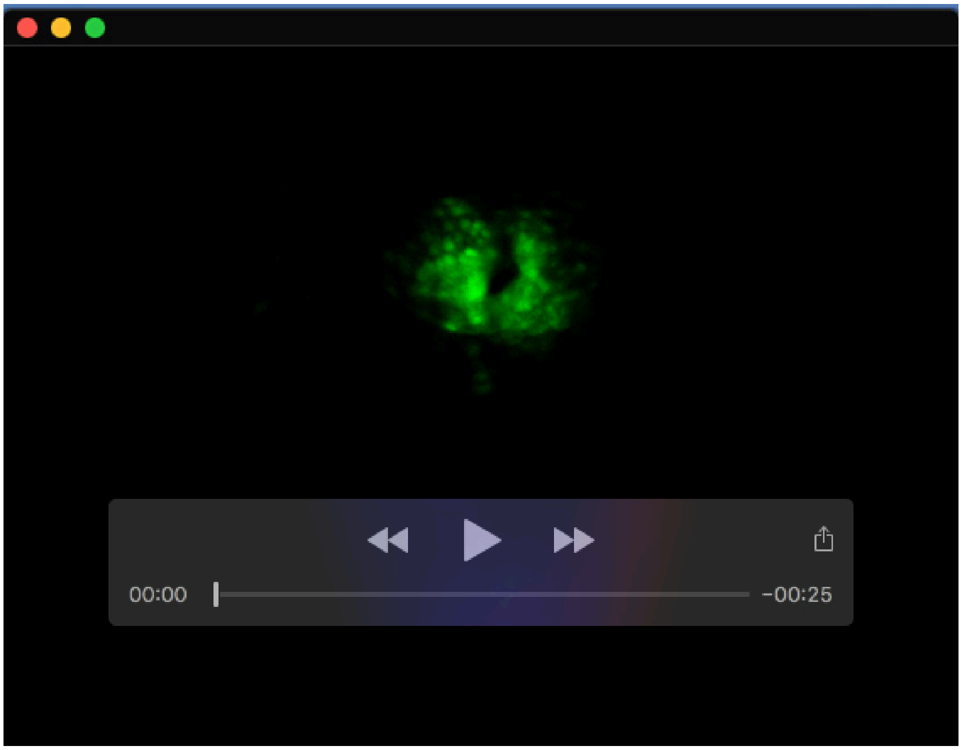

Movie 2. Live imaging of CPCs jogging in a *ntl* mutant *Tg(myl7:EGFP)* embryo from the onset of cardiac cone formation until three hours after.

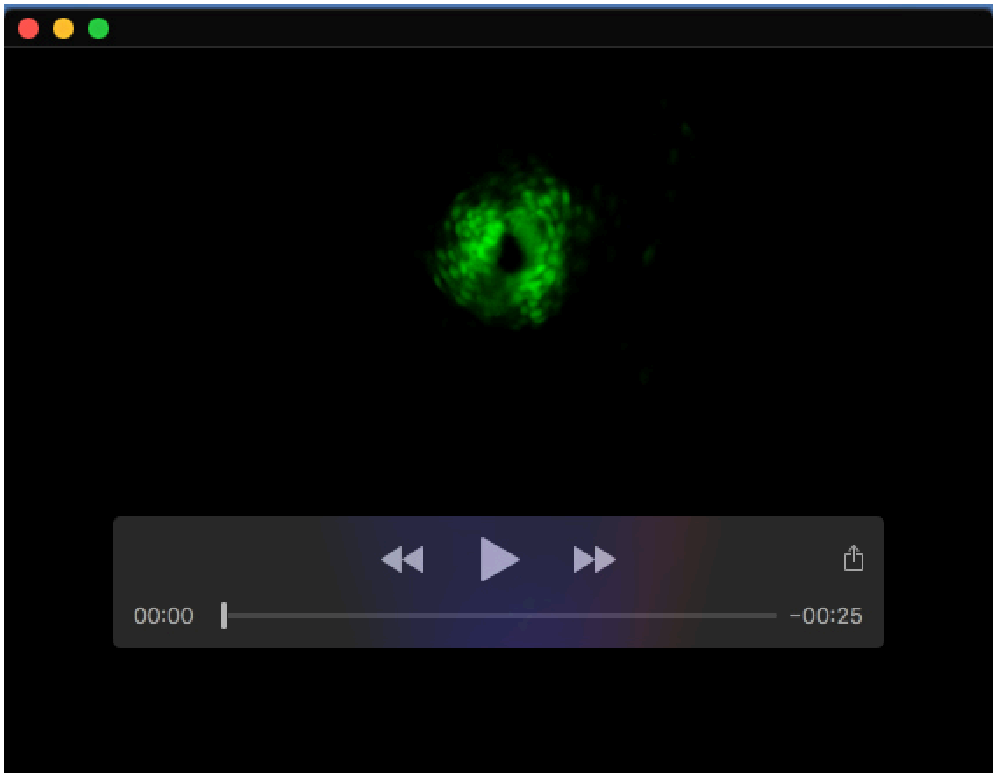

Movie 3. Live imaging of CPCs jogging in a *spaw* mutant *Tg(myl7:EGFP)* embryo from the onset of cardiac cone formation until three hours after.

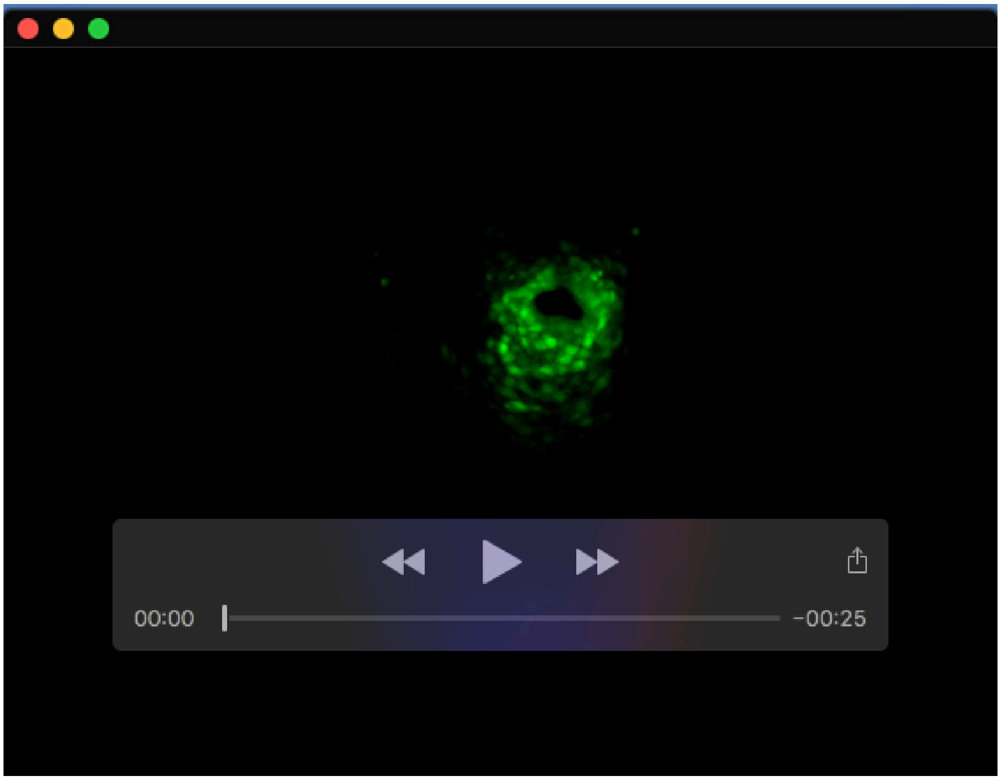

Movie 4. Live imaging of CPCs jogging in a SU5402-treated *Tg(myl7:EGFP)* embryo from the onset of cardiac cone formation until three hours after.

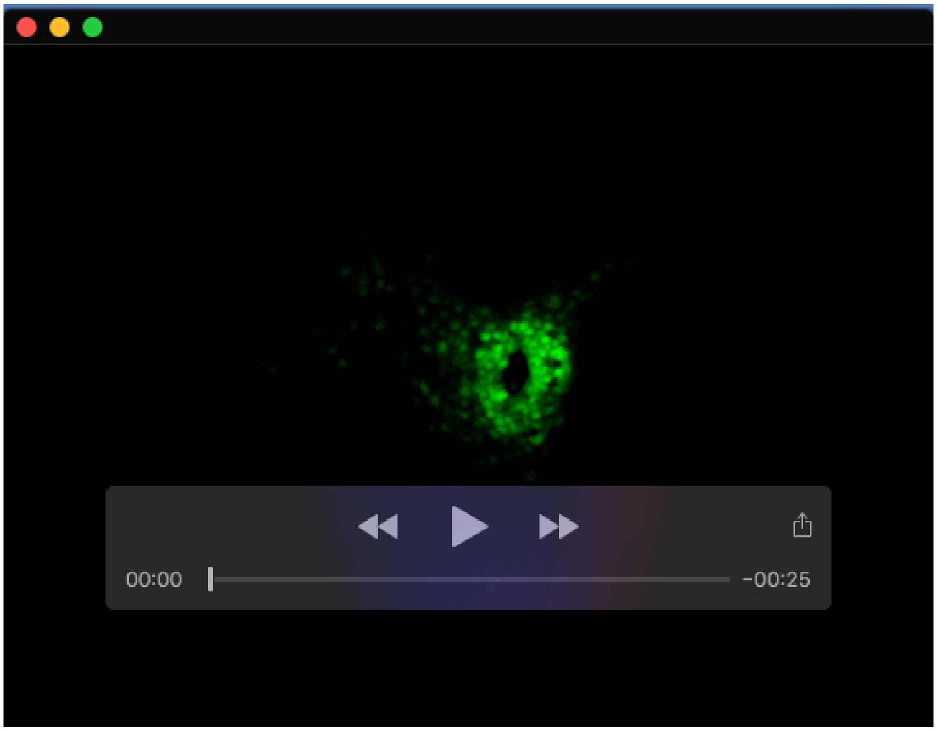

Movie 5. Live imaging of CPCs jogging in a *spaw* mutant and SU5402-treated *Tg(myl7:EGFP)* embryo from the onset of cardiac cone formation until three hours after.

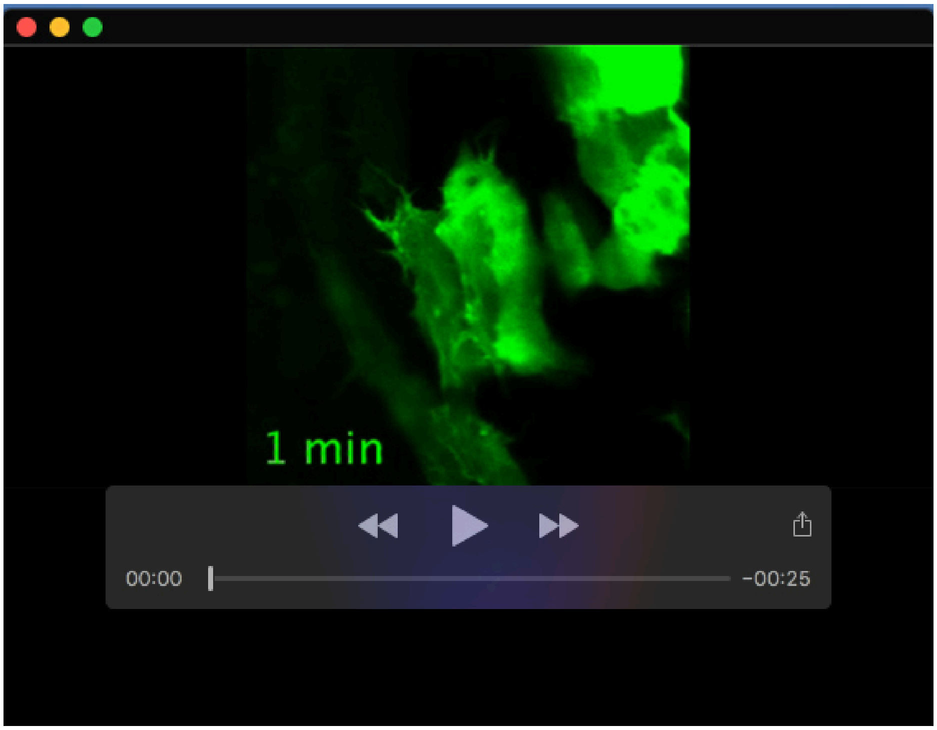

Movie 6. Live imaging of F-actin in a singular cardiac progenitor cell (CPC) from a WT mosaic *Tg(myl7:Lifeact-EGFP)* embryo at 21 hours post fertilization (hpf). Movie is a total of 5 minutes.

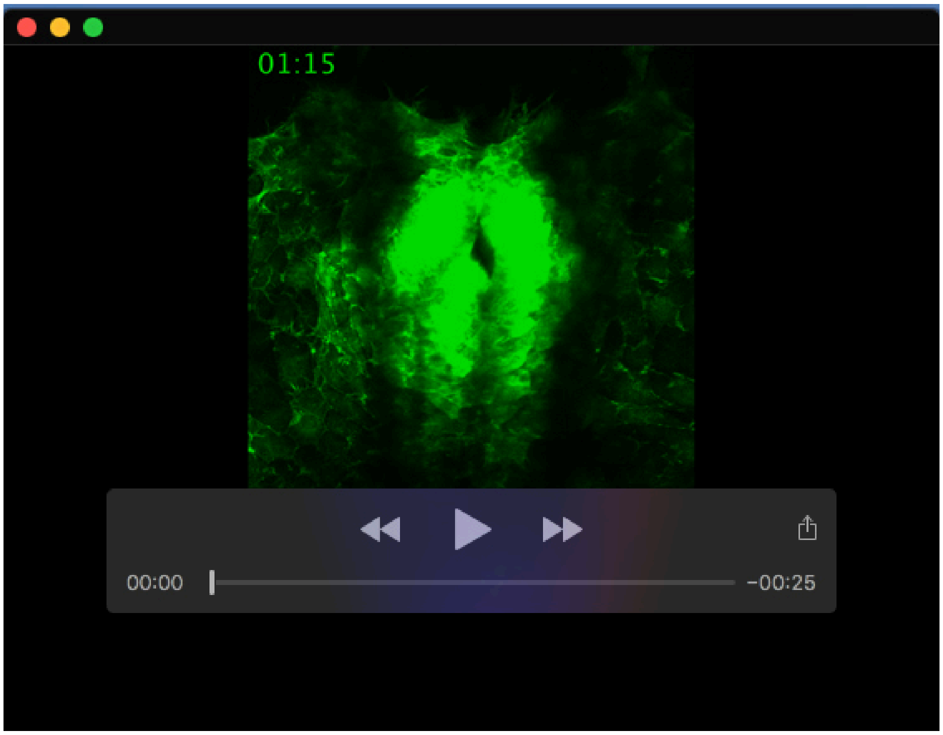

Movie 7. Live imaging of F-actin in a WT transgenic *Tg(myl7:Lifeact-EGFP)* embryo from 19-23 hpf, following the cardiac cone as it undergoes jogging.

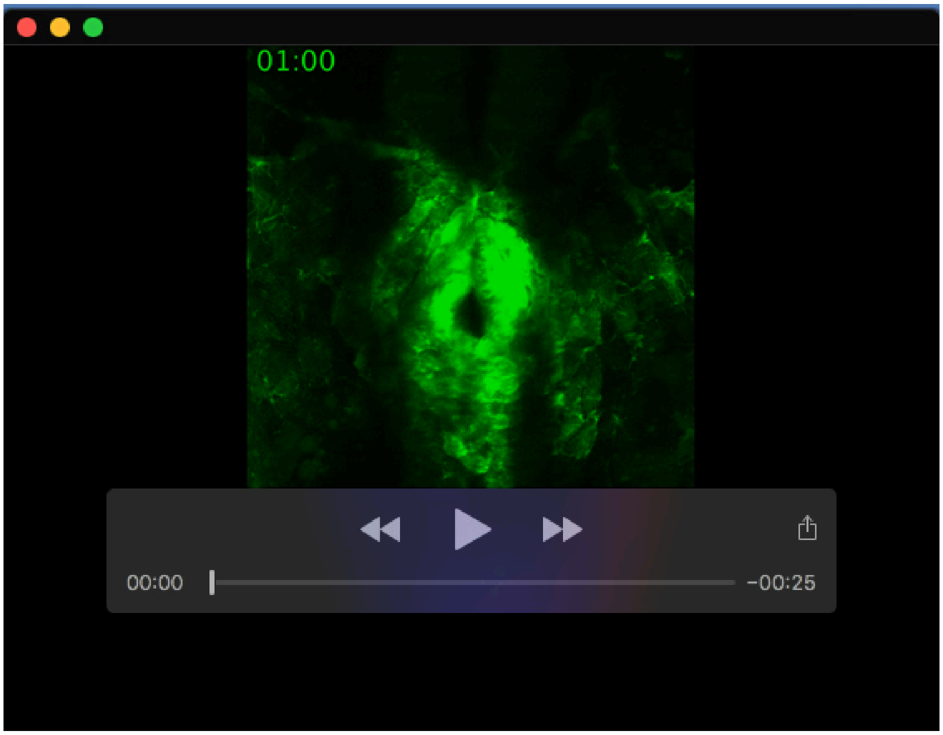

Movie 8. Live imaging of F-actin in a *spaw* morphant *Tg(myl7:Lifeact-EGFP)* embryo from 19-23 hpf, following the cardiac cone as it undergoes jogging.

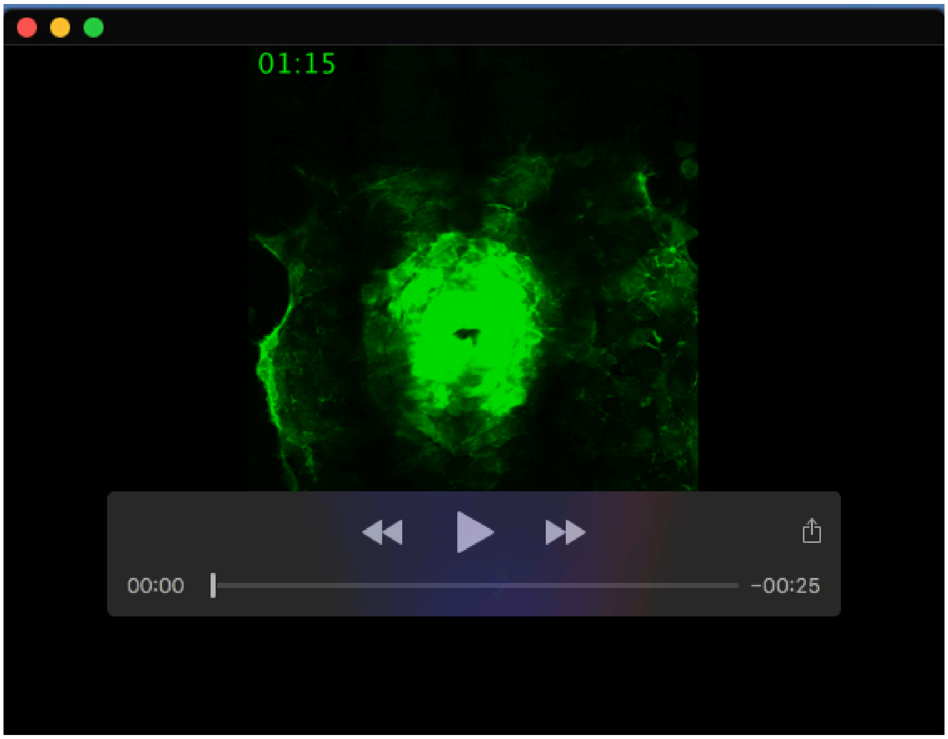

Movie 9. Live imaging of F-actin in a SB-505124-treated *Tg(myl7:Lifeact-EGFP)* embryo from 19-23 hpf, following the cardiac cone as it undergoes jogging.

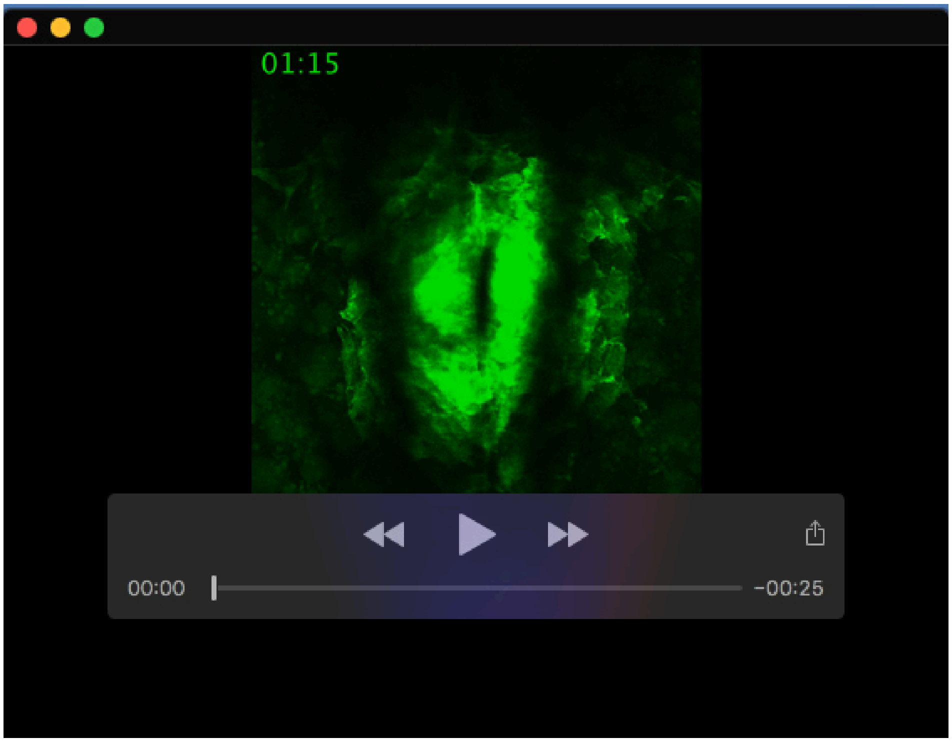

Movie 10. Live imaging of F-actin in a *ntl* morphant *Tg(myl7:Lifeact-EGFP)* embryo from 19-23 hpf, following the cardiac cone as it undergoes jogging.
